# Antioxidant and Anti-Inflammatory Properties of Longan (*Dimocarpus longan* Lour.) Pericarp

**DOI:** 10.1155/2012/709483

**Published:** 2012-08-26

**Authors:** Guan-Jhong Huang, Bor-Sen Wang, Wei-Chao Lin, Shyh-Shyun Huang, Chao-Ying Lee, Ming-Tsung Yen, Ming-Hsing Huang

**Affiliations:** ^1^School of Chinese Pharmaceutical Sciences and Chinese Medicine Resources, College of Pharmacy, China Medical University, Taichung 402, Taiwan; ^2^Department of Food Science and Technology, Chia Nan University of Pharmacy and Science, 60 Erh-Jen Road, Section 1, Jen-Te, Tainan 717, Taiwan; ^3^Department of Applied Life Science and Health, Chia Nan University of Pharmacy and Science, 60 Erh-Jen Road, Section 1, Jen-Te, Tainan 717, Taiwan; ^4^Department of Cosmetic Science, Chia Nan University of Pharmacy and Science, 60 Erh-Jen Road, Section 1, Jen-Te, Tainan 717, Taiwan; ^5^School of Pharmacy, College of Pharmacy, China Medical University, Taichung 404, Taiwan

## Abstract

This study examined the antioxidant and anti-inflammatory activities of the water extract of longan pericarp (WLP). The results showed that WLP exhibited radical scavenging, reducing activity and liposome protection activity. In addition, WLP also inhibited lipopolysaccharide (LPS)-induced nitric oxide (NO) production in macrophages. Further, administration of WLP, in the range of 100–400 mg/kg, showed a concentration-dependent inhibition on paw edema development following carrageenan (Carr) treatment in mice. The anti-inflammatory effects of WLP may be related to NO and tumor necrosis factor (TNF-**α**) suppression and associated with the increase in the activities of antioxidant enzymes, including catalase, superoxide dismutase, and glutathione peroxidase. Overall, the results showed that WLP might serve as a natural antioxidant and inflammatory inhibitor.

## 1. Introduction

Inflammation has been recognized as a localized protective reaction of tissue to injury or infection that is characterized by pain, redness, and swelling. The inflammatory process involves multiple physiological systems with the immune system playing a central role [1]. Chronic inflammation results in upregulation of many enzymes and signaling proteins in affected areas. These proinflammatory enzymes include the inducible forms of nitric oxide synthase (iNOS) and cyclooxygenase-2 (COX-2). The iNOS and COX-2 are responsible for elevated levels of nitric oxide (NO) and prostaglandins (PGs), respectively [2]. A previous study indicated that the chronic inflammation correlates with an increase in iNOS activity [3]. The most significant evidence for NO as a mediator of tissue injury has been obtained from studies on an animal arthritis model, human osteoarthritis, and rheumatoid arthritis [4]. Further, intracellular protective mechanisms against these inflammatory stresses involve antioxidant enzymes including superoxide dismutase (SOD), catalase (CAT), and glutathione peroxidase (GPx) in tissues. Recently, it has been shown that faulty cellular antioxidant systems cause organisms to develop a series of inflammatory and cancer diseases [5]. It appears that the important roles of enzymatic antioxidants protect organisms against oxidative stress in the process of inflammation [6]. This has triggered studies focusing on the role of natural products in suppressing the production of oxidative stress and increasing enzymatic antioxidants in tissues. 

Longan (*Dimocarpus longan *Lour.) is widely distributed in Southeast Asia, such as China, Taiwan, Vietnam, and Thailand. Its fruit is accepted by consumers over the world due to its sweet and juicy sensation in the mouth and health benefits [7]. The fruit of *Dimocarpus longan* was used as a traditional Chinese medicine for different treatments, such as promoting blood metabolism, soothing nerves, and relieving insomnia [8]. Being considered as a food in the process of fresh fruit or processing longan products, longan pericarp tissues act as agricultural wastes and create environmental problems at a large scale. Additionally, longan pericarp tissues contain high amounts of bioactive compounds, such as phenolic acids, flavonoids, and polysaccharides [9, 10], and exhibit antibacterial, antiviral, antioxidant, anti-inflammatory, and anticarcinogenic properties [7, 11, 12]. Although the pericarp of *Dimocarpus longan* has shown some physiological effects, there are no studies focusing on their effects on the anti-inflammatory property* in vitro and in vivo* so far as we know. In this study, we evaluated the anti-inflammatory effects of WLP using lipopolysaccharide (LPS)-stimulated mouse RAW264.7 macrophages *in vitro* and carrageenan (Carr)-induced mouse paw edema model *in vivo*. 

## 2. Materials and Methods

### 2.1. Materials

Glutathione (GSH), 1,1-diphenyl-2-picrylhydrazyl radicals (DPPH), lipopolysaccharide (LPS; endotoxin from *Escherichia coli*, serotype 0127:B8), 1,1-diphenyl-2-picrylhydrazyl (DPPH), 2,2′-azinobis-(3-ethylbenzothiazoline)-6-sulphonic acid (ABTS), butylated hydroxytoluene (BHT), 3-[4,5-dimethylthiazol-2-yl]-2,5-diphenyltetrazolium bromide (MTT), *λ*-Carrageenan (Carr), and indomethacin (Indo) were obtained from Sigma-Aldrich (St. Louis, MO, USA). Deionized water from a Milli-Q system (Millipore, Bedford, MA, USA) was used to prepare all buffers and sample solutions. *Dimocarpus longan* fruits of cultivar “Feng Ko” were harvested from a commercial orchard in Taichung, identified and authenticated by Dr. Shyh-Shyun Huang of the Institute of Chinese Pharmaceutical Science, China Medical University, Taichung, Taiwan. The purity of three marker standards, gallic acid, epicatechin, and ellagic acid was judged by a photodiode array detector (Hitachi L-7455). Butyl p-hydroxybenzoate was an internal standard (IS).

### 2.2. Sample Preparation

The pericarps were separated from the whole fresh longanfruits by hand, and then ground after drying in oven at 55 ± 0.5°C for 12 hours. The powder (100 g) was extracted with water (1000 mL) at 100°C for 60 min and then centrifuged at 10,000 ×g for 20 min. The extract was filtered, and the residue was reextracted under the same conditions. The combined filtrate was then freeze dried. The yield obtained was 12.9% (w/w). The final sample was named as water extract of longan pericarp (WLP). A voucher specimen is deposited at the department of cosmetic Science, Chia Nan University of Pharmacy and Science, Tainan, Taiwan.

### 2.3. High-Performance Liquid Chromatography (HPLC) Analysis

HPLC was performed with a Hitachi Liquid Chromatograph (Hitachi Ltd., Tokyo, Japan), consisting of two model L-7100 pumps, and one model L-7455 photodiode array detector (254 nm). Samples of WLP (10 mg/mL) were filtered through a 0.45 *μ*m filter and injected into the HPLC column. The injection volume was 10 *μ*L, and the flow rate was 0.8 mL/min. The separation temperature was 25°C. The column was a Mightysil RP-18 GP (5 *μ*m, 250 × 4.6 mm I.D.; Kanto Corporation, Portland, OR, USA). The method involved the use of a binary gradient with mobile phases containing (a) phosphoric acid in water (0.1%, v/v) and (b) H_2_O/CH_3_CN (2 : 8, v/v). The solvent gradient elution program was as follows: 0–10 min, 100–95% A, 0–5% B; 10–20 min, 95–85% A, 5–15% B; 20–30 min, 85–70% A, 15–30% B; 30–40 min, 70–50% A, 30–50% B; 40–50 min, 50–0% A, 50–100% B; finally 50–70 min, 0% A, 100% B.

### 2.4. Determination of Total Polyphenols

Total polyphenols were determined as gallic acid equivalents [13]. Two mL of sodium carbonate (20% (w/v)) was added to different concentrations of samples in a 10 mL volumetric flask. After 5 min, 0.1 mL of Folin-Ciocalteu reagent (50% (v/v)) was added, and the volume increased to 10 mL with H_2_O. After incubation at 30°C for 1 h, the absorbance was measured at 750 nm and compared to a gallic acid calibration curve. 

### 2.5. Determination of ABTS Radical Inhibition

This assay determined the capacity of samples to scavenge ABTS^•+^ as previously described [14]. The ABTS^•+^ was generated by reacting 1 mM ABTS with 0.5 mM hydrogen peroxide and 10 units/mL horseradish peroxidase in the dark at 30°C for 2 h. After 1 mL of ABTS^•+^ was added to samples, the absorbance at 734 nm was recorded after 10 min. A lower level of absorbance indicated better radical scavenging activity. 

### 2.6. Determination of DPPH Radical Inhibition

The effects of samples on the DPPH radical were estimated according to the method described in a previous study [15]. The samples were added to a methanolic solution (1 mL) of DPPH radical (the final concentration of DPPH was 0.2 mM). The mixture was shaken vigorously and allowed to stand at room temperature for 30 min; the absorbance of the resulting solution was then measured spectrophotometrically at 517 nm.

### 2.7. Determination of Reducing Activity

The reducing power of sample was determined as previously described [16]. Potassium ferricyanide (2.5 mL, 10 mg/mL) was added to samples in phosphate buffer (2.5 mL, 200 mM, pH 6.6) and the mixture was incubated at 50°C for 20 min. Trichloroacetic acid (2.5 mL, 100 mg/mL) was added to the mixture, which was then centrifuged at 1,000 g for 10 min. The supernatant (2.5 mL) was mixed with distilled water (2.5 mL) and ferric chloride (0.5 mL, 1.0 mg/mL), and then the absorbance was read at 700 nm. Higher absorbance of the reaction mixture indicated greater reducing activity. 

### 2.8. Determination of Liposome Oxidation

Lecithin (500 mg) was sonicated in an ultrasonic cleaner (Branson 8210, Branson ultrasonic Corporation, Danbury, CT, USA) in phosphate buffer (50 mL, 10 mM, pH 7.4) for 2 h in an ice-cold water bath. The sonicated solution, FeCl_3_, ascorbic acid, and samples (0.2 mL) were mixed to produce a final concentration of 3.12 *μ*M FeCl_3_, and 125 *μ*M of ascorbic acid. The mixture was incubated for 1 h at 37°C. The liposome oxidation was determined by the thiobarbituric acid (TBA) method. The absorbance of the sample was read at 532 nm against a blank, which contained all reagents except lecithin. A lower level of absorbance indicated stronger protective activity. 

### 2.9. Animals

Male ICR mice (6–8 weeks) were obtained from the BioLASCO Taiwan Co., Ltd. (Taipei, Taiwan). The animals were kept in plexiglass cages at a constant temperature of 22 ± 1°C, and relative humidity of 55 ± 5% with 12 h dark-light cycle for at least 2 weeks before the experiment. Animals were given food and water *ad libitum*. All experimental procedures were performed according to the National Institutes of Health Guide for the Care and Use of Laboratory Animals. 

### 2.10. *λ*-Carrageenan (Carr)-Induced Edema

Carr-induced hind paw edema model was used to determine anti-inflammatory activity [17]. After a 2-week adaptation period, mice (18–25 g) were randomly divided into five groups (*n* = 8 in each group). (1) Carr alone group: mice were injected with 1% Carr (50 *μ*L) in the plantar side of right hind paws. (2) Positive indomethacin (Indo) control group: Indo (10 mg/kg) was orally administered 90 min before the injection of Carr. (3–5) WLP-treated groups: WLP was administered orally at a dose of 100, 200, and 400 mg/kg for 2 h before the injection Carr. Paw volume was measured immediately after Carr injection at 1, 2, 3, 4, and 5 h intervals using a plethysmometer (model 7159, Ugo Basile, Varese, Italy). The degree of swelling induced was evaluated by the ratio *a- b*, where *a* is the volume of the right hind paw after Carr treatment, and *b* was the volume of the right hind paw before Carr treatment. After 5 h, the animals were sacrificed, and the right hind paw tissue was dissected. The right hind paw tissue was rinsed in ice-cold normal saline, and immediately placed in cold normal saline four times their volume and homogenized at 4°C. Then the homogenate was centrifuged at 12,000 ×g for 5 min. The supernatant was obtained for tissue lipid peroxidation assays and antioxidant enzymes activity assays. Also, blood was withdrawn for serum NO and TNF-*α* assay.

### 2.11. Determination of Lipid Peroxidation in Edema Paws

The hind paw tissue lipid oxidation was evaluated by the thiobarbituric acid (TBA) method. Briefly, lipid degradation products reacted with thiobarbituric acid in the acidic high temperature and formed red-complex TBARS. The absorbance of TBARS was determined at 532 nm.

### 2.12. Measurement of Serum TNF-*α* Levels

Serum levels of TNF-*α* were determined using a commercially available ELISA kit according to the manufacturer's instruction. TNF-*α* was determined from a standard curve. The concentrations were expressed as pg/mL.

### 2.13. Antioxidant Enzyme Activity Measurements

Total SOD activity was determined by the inhibition of cytochrome *c* reduction [18]. The reduction of cytochrome *c* was mediated by superoxide anions generated by the xanthine/xanthine oxidase system and monitored at 550 nm. One unit of SOD was defined as the amount of enzyme required to inhibit the rate of cytochrome *c* reduction by 50%. Total CAT activity was measured as previously described [19]. In brief, the reduction of 10 mM H_2_O_2_ in 20 mM of phosphate buffer (pH 7.0) was monitored by measuring the absorbance at 240 nm. Total GPx activity in cytosol was determined according to the method described in a previous study [20]. The enzyme solution was added to a mixture containing hydrogen peroxide and glutathione in 0.1 mM Tris buffer (pH 7.2), and the absorbance at 340 nm was measured. Activity was evaluated from a calibration curve, and the enzyme activity was defined as nanomoles of NADPH oxidized per milligram protein per minute.

### 2.14. Cell Culture

A murine macrophage cell line RAW264.7 was purchased from Food Industry Research and Development Institute (Hsinchu, Taiwan). Cells were cultured in plastic dishes containing Dulbecco's Modified Eagle Medium (DMEM, Sigma, St. Louis, MO, USA) supplemented with 10% fetal bovine serum (FBS, Sigma) in a CO_2_ incubator (5% CO_2_) at 37°C.

### 2.15. Cell Viability

Cells (2 × 10^5^) were cultured in 96-well plate containing DMEM supplemented with 10% FBS for 1 day. Then cells were cultured with samples in the presence of 100 ng/mL LPS (lipopolysaccharide) for 24 h. After that, the cells were washed twice with PBS and incubated with 100 *μ*L of 0.5 mg/mL MTT for 2 h at 37°C testing for cell viability. The medium was then discarded, and 100 *μ*L dimethyl sulfoxide (DMSO) was added. After 30-min incubation, absorbance at 570 nm was read using a microplate reader.

### 2.16. Measurement of Nitric Oxide/Nitrite

Nitrite levels in the cultured media and serum, which reflect intracellular nitric oxide synthase activity, were determined by Griess reaction. The cells were incubated with samples in the presence of LPS (100 ng/mL) at 37°C for 24 h. Then, cells were dispensed into 96-well plates, and 100 *μ*L of each supernatant was mixed with the same volume of Griess reagent (1% sulfanilamide, 0.1% naphthyl ethylenediamine dihydrochloride, and 5% phosphoric acid) and incubated at room temperature for 10 min. Using sodium nitrite to generate a standard curve, the concentration of nitrite was measured from absorbance at 540 nm [17].

### 2.17. Western Blot Analysis

Total protein was extracted with a RIPA solution (radio-immuno-precipitation assay buffer) at −20°C overnight. Protein samples (30 *μ*g) were resolved by denaturing sodium dodecyl sulfate-polyacrylamide gel electrophoresis (SDS-PAGE) using standard methods and then were transferred to PVDF membranes by electroblotting and blocking with 1% BSA. The membranes were probed with the primary antibodies (iNOS, COX-2, and *β*-actin) at 4°C overnight, washed three times with PBST, and incubated for 1 h at 37°C with horseradish peroxidase conjugated secondary antibodies. The membranes were washed three times, and the immunoreactive proteins were detected by enhanced chemiluminescence (ECL) using hyperfilm and ECL reagent (Amersham International plc., Buckinghamshire, UK). The results of Western blot analysis were quantified by measuring the relative intensity compared to the control using Kodak Molecular Imaging Software and represented in the relative intensities.

### 2.18. Statistical Analysis

Statistical evaluation was carried out by one-way analysis of variance (ANOVA followed by Scheffe's multiple range test). Statistical significance is expressed as *P < 0.05, **P < 0.01, and ***P < 0.001.

## 3. Results 

### 3.1. Fingerprint Chromatogram of WLP by HPLC

**Figure 1:**
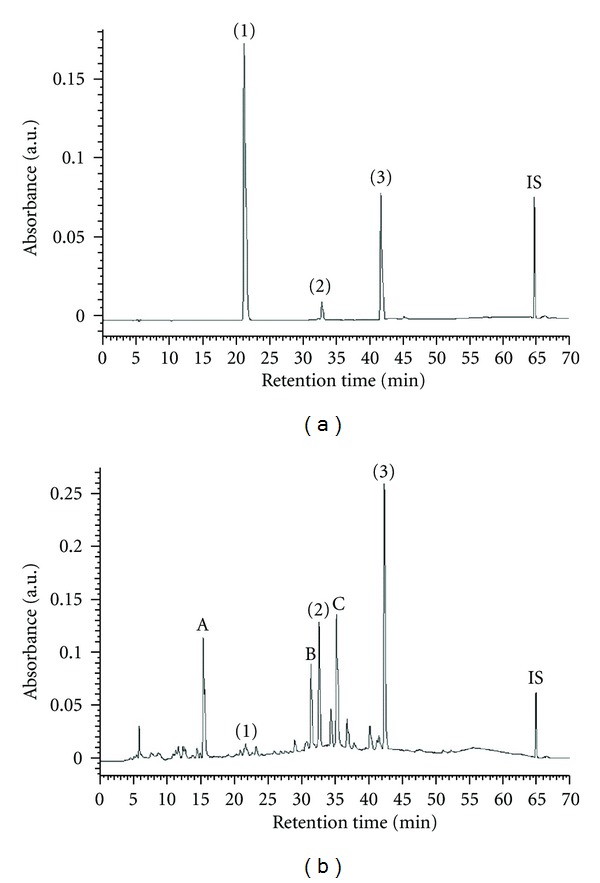
HPLC chromatograms of three markers (a) and water extract of longan pericarp (b) (1) gallic acid; (2) epicatechin; (3) ellagic acid; IS: butyl p-hydroxybenzoate.

To establish the fingerprint chromatogram of WLP, gallic acid, epicatechin, and ellagic acid were used as markers. As shown in Figure 1, these phenolic components were identified as gallic acid, epicatechin, and ellagic acid. Butyl *p*-hydroxybenzoate was an internal standard (IS). Based on the plots of the peak-area ratio (*y*) versus concentration (*x*, *μ*g/mL), the regression equations of the three phenolic constituents and their correlation coefficients (*r* ^2^) were as follows: gallic acid, *y* = 0.0588*x* + 0.2488 (*r* ^2^ = 0.9961); epicatechin, *y* = 0.0036*x* + 0.0597 (*r* ^2^ = 0.9973); ellagic acid, *y* = 0.0249*x* + 0.1539 (*r* ^2^ = 0.9995). The percentagerecoveryforgallic acid, epicatechin, and ellagic acid was 98.5%, 100.3%, and 99.8%, respectively (each spikedwith 10 ppm). The relative amounts of these three phenolic compounds identified in WLP were in the order of ellagic acid (26.5 mg/g extract) > epicatechin (8.1 mg/g extract) > gallic acid (0.7 mg/g extract). On the other hand, in Figure 1(b), three main peaks from unknown compounds A, B, and C with retention time at 15.5 min, 31.3 min, and 35.1 min, respectively, were observed. By ultraviolet-visible spectra analysis, compounds A, B, and C showed the absorbance maximum wavelength at 214.8 and 271.0 nm; 218.6 and 275.7 nm; 253.1 nm, respectively. Peaks A, B, and C could be recognized as characteristic peaks of WLP on the basis of their retention time and UV spectra. However, the HPLC analyses showed that gallic acid, epicatechin, and ellagic acid, which are known with bioactive actions, were found in WLP.

### 3.2. The Contents of Total Phenols and the Antioxidant Activities of WLP

The total phenol contents in the WLP were determined in gallic acid equivalents (Table 1). The results showed that WLP with 200 *μ*g of dry extract/mL contained amounts of total phenols equal to 29.2 mg gallic acid/mL. In other words, the level of polyphenolic was 146.0 mg (gallic acid equivalents) in each gram of WLP extract. Table 1 also shows ABTS scavenging, DPPH scavenging, reducing power, and liposome protection of the WLP. WLP showed 46.6–94.8% and 36.0–96.7% scavenging activity on ABTS radicals and DPPH radical scavenging in the range of 50–200 *μ*g/mL. The reducing activity of natural products can usually be achieved by terminating the radicals' chain reaction. The reducing activity of WLP occurred in a concentration-dependent manner and increased to 2.2-fold in the range of 50–200 *μ*g/mL. In addition, as shown in Table 1, liposome protection was used as an index to assay the protective activity of the WLP on lipid molecules. WLP in the range of 50–200 *μ*g/mL exhibited a dose dependently protective effect, 12.6–37.0%, on the liposome damage induced by the Fe^3+^/H_2_O_2_ reaction. These data imply that the antioxidant properties of WLP could protect lipid molecules against oxidative damage.

### 3.3. Effects of WLP and Its Reference Compounds on LPS-Induced NO Production in RAW264.7 Macrophages

**Figure 2:**
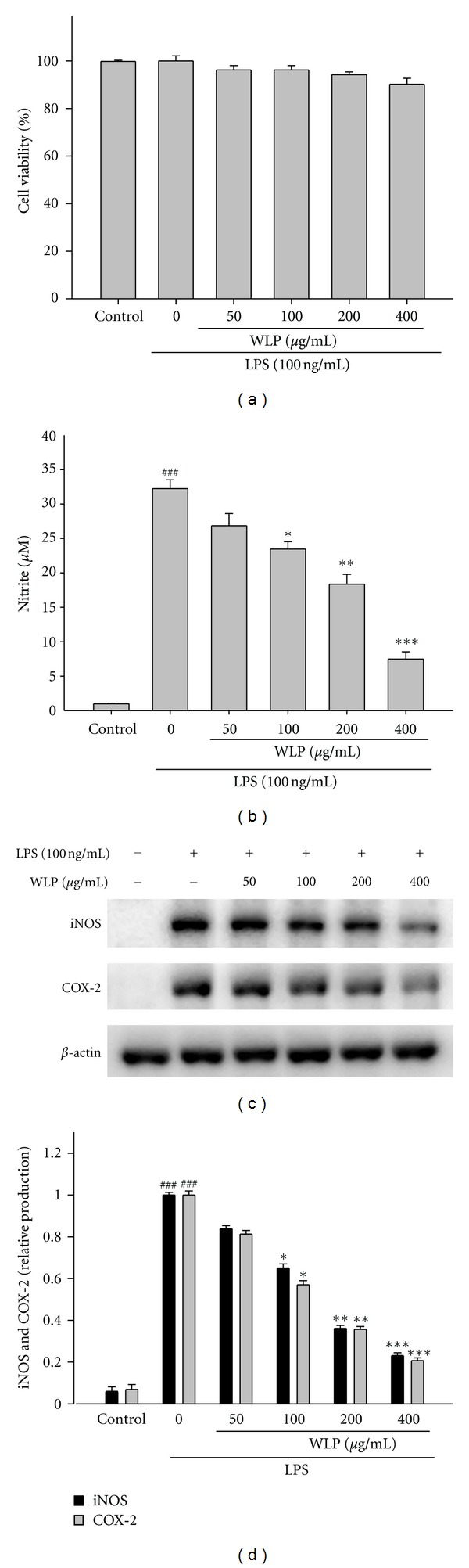
Effects of WLP on cell viability (a), NO production (b), iNOS and COX-2 protein expressions (c), and relative iNOS and COX-2 protein levels (d) in lipopolysaccharide (LPS)-stimulated RAW264.7 cells. The data were presented as mean ± SEM for three different experiments performed in triplicate. ^###^ *P* < 0.001 as compared with control group. *P < 0.05, **P < 0.01, and ***P < 0.001 as compared with LPS alone group.

In a cellular model of inflammation, the NO inhibitory activity of the WLP was determined by using the LPS-activated macrophages to produce NO radicals that were measured as nitrites in the culture medium by the Griess reaction. In this study, WLP reduced the NO production of activated macrophages with an IC_50_ value of 179.8 *μ*g/mL. This suggests that WLP could be a potential inhibitor of NO-related inflammation pathway. In addition, no cell toxicity was observed with WLP, as measured by the MTT cell viability test (Figure 2(a)). In addition, gallic acid and ellagic acid showed the NO inhibitory activity induced by LPS in RAW264.7 macrophages with an IC_50_ value of 18.8 and 5.2 *μ*g/mL, respectively. Epicatechin had weak NO inhibitory activity induced by LPS in RAW264.7 macrophages. 

### 3.4. Effects of WLP on LPS-Induced Cell Viability, iNOS, and COX-2 Expressions

WLP at doses of 50, 100, 200, and 400 *μ*g/mL decreased the NO levels (Figure 2(b)) in RAW264.7 macrophages. In addition, WLP at doses of 100, 200, and 400 *μ*g/mL significantly inhibited LPS-induced iNOS and COX-2 protein expressions in RAW264.7 macrophage cells (Figures 2(c) and 2(d)). Further experiments were conducted to determine the NO production from WLP, gallic acid, epicatechin, and ellagic acid. Our results demonstrate that WLP, gallic acid, and ellagic acid significantly inhibited NO production. Inhibition of NO production (IC_50_) in LPS-induced RAW264.7 macrophages by WLP and its reference compounds was demonstrated as WLP (179.8 ± 1.4 *μ*g/mL), gallic acid (18.8 ± 0.3 *μ*g/mL), ellagic acid (5.2 ± 0.2 *μ*g/mL), and indomethacin (Indo) (52.5 ± 2.7 *μ*g/mL) (Table 2).

### 3.5. *λ*-Carrageenan (Carr)-Induced Edema

**Figure 3:**
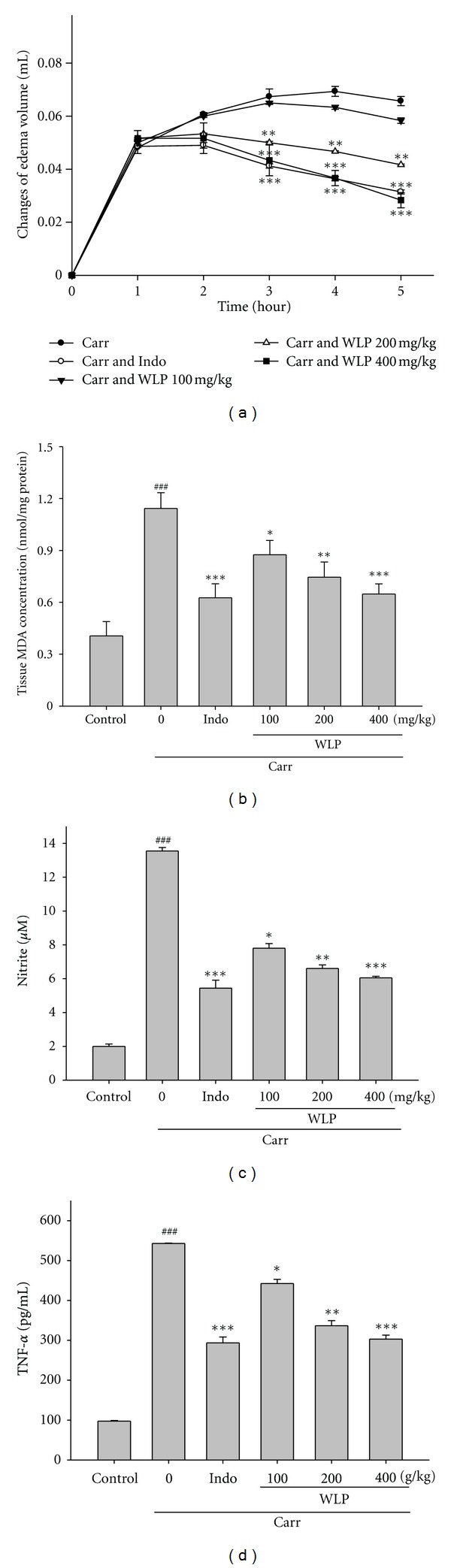
Effects of WLP and Indo on hind paw edema (a), the tissue MDA concentration of foot (b), serum NO (c), and TNF-*α* (d) concentrations of serum at the 5th h in Carr-induced mice. The data were presented as mean ± SEM for three different experiments performed in triplicate. ^###^ *P* < 0.001 as compared with control group. *P < 0.05, **P < 0.01, and ***P < 0.001 as compared with Carr alone group.

According to Figure 3(a), Indo reduced the edema volumes by 0–39% in comparison to the Carr alone group at 1–5 h after Carr treatment. On the other hand, in the range of 100–400 mg/kg, WLP showed a concentration-dependent inhibition of edema development following Carr treatment. For WLP at the concentration of 400 mg/kg, the levels of edema volume were decreased to 58% of that observed in the Carr alone group after 5 h treatment. There was no significant difference between the Indo (10 mg/kg) and WLP (400 mg/kg) groups at 1–5 h after Carr treatment (Figure 3(a)). Meanwhile, the tissue MDA level was significantly decreased by treatment with WLP (400 mg/kg), as well as 10 mg/kg Indo 5 h after Carr injection (Figure 3(b)). The inhibition of MDA production compared with the Carr group is 23%, 35%, and 44% at the 100, 200, and 400 mg/kg of WLP, respectively.

 Many studies have demonstrated evidence for increased NO, enhanced iNOS expression, and elevated TNF-*α* production in inflammatory processes. The level of nitrites in serum is a regular index for intracellular NO and iNOS production *in vivo.* WLP (100, 200, and 400 mg/kg) significantly (*P* < 0.05, *P* < 0.01, and *P* < 0.001) decreased the NO levels in serum (Figure 3(c)) at the 5th h after Carr injection. Indo (10 mg/kg) significantly decreased the NO level in serum at the 5th h after Carr injection (*P* < 0.001). On the other hand, WLP (100, 200, and 400 mg/kg) decreased the TNF-*α* level in serum at the 5th h after Carr injection (Figure 3(d)). Indo (10 mg/kg) significantly decreased the TNF-*α* level in serum at the 5th h after Carr injection (*P* < 0.001). The inhibition rates of TNF-*α* levels compared with the Carr group are 19%, 38%, and 45% at the 100, 200, and 400 mg/kg of WLP, respectively. 

**Figure 4:**
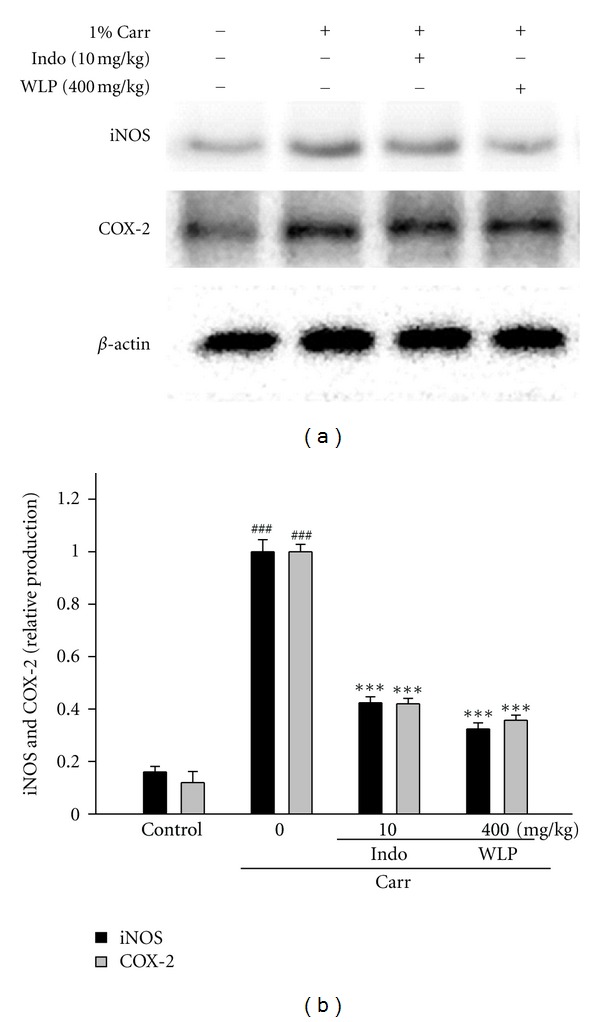
Inhibition of iNOS and COX-2 protein expression of foot by WLP at the 5th hr in Carr-induced mice (a) *β*-Actin was used as an internal control. Relative iNOS and COX-2 protein levels were calculated with reference to Carr group (b) The data were presented as mean ± SEM for three different experiments performed in triplicate.^ ###^ *P* < 0.001  as compared with control group. ***P < 0.001 as compared with Carr alone group.

Under healthy conditions, antioxidant enzymes decrease the accumulation of harmful oxidative stress. The activities of CAT, SOD, and GPx in the livers of Carr-induced mice were determined. In the range of 100–400 mg/kg, WLP increased the activities of CAT to 121%–144%, SOD to 128%–153%, and GPx to 111%–126% of that observed in the Carr alone group. Indo also exhibited increased effects on the activities of CAT (141%), SOD (161%), and GPx (116%) compared to the Carr alone group. These data imply that the protective effects of WLP might be attributed to its elevation of antioxidant enzymes activities in Carr-induced inflammation (Table 3). Meanwhile, according to Figure 4(a), the results showed that injection of WLP (400 mg/kg) on Carr induced for 5th h inhibited iNOS and COX-2 proteins expression in mouse paw tissues. The intensities of the protein bands showed an average of 68% and 65% downregulation of iNOS and COX-2 protein, respectively, after treatment with WLP compared with the Carr induced alone (Figure 4(b)). In addition, the protein expression showed an average of 58% and 59% downregulation of iNOS and COX-2 protein after treatment with Indo at 10.0 mg/kg compared with the Carr induced alone. 

## 4. Discussion 

The DPPH and ABTS assays have been a popular radical scavenging test for natural ingredients. Free radicals could induce biological damage and pathological events, such as inflammation, aging, and carcinogenesis [21]. In this study, WLP showed significant antioxidant activity and could be a natural free radical inhibitor (Table 1). By HPLC analysis, three active phenolic components in WLP were identified as gallic acid, epicatechin, and ellagic acid which have been reported as important constituents of longan pericarp [9]. Gallic acid and ellagic acid showed significant inhibition of NO production. Gallic acid inhibited histamine release and proinflammatory cytokine production in mast cells [22]. Gallic acid also suppresses LPS-induced nuclear factor-kappaB signaling in A549 lung cancer cells [23]. On the other hand, ellagic acid inhibited oxidized LDL-mediated LOX-1 expression, ROS generation, and inflammation in human endothelial cells [24]. Ellagic acid also inhibited LPS-induced expression of enzymes involved in the synthesis of prostaglandin E2 in human monocytes [25]. Ellagic acid found in WLP also showed significant antioxidant effects by various antioxidation assays [26]. Thus, ellagic acid exhibited the high efficiency in WLP, which can benefit the pharmaceutical and food industry. We also observed that the total polyphenols of WLP are closely correlated with its radicals scavenging activity. In addition, liposome was prepared from phospholipids and used to imitate the lipid oxidation of biomolecules. In this study, WLP displayed protective activity against lipid oxidation, indicating that WLP could protect biolipid molecules from oxidative damage *in vivo*. Our results are in agreement with other authors who have reported parallel findings including DPPH radical scavenging activity and lipid peroxidation inhibitory activity [27]. According to Table 1, the data implies that the total polyphenol of WLP could contribute to the radical scavenging and reducing activities, as well as lipid protection. 

The L-arginine-NO pathway has been proposed to play an important role in the Carr-induced inflammatory response [28]. The expression of the inducible isoform of NO synthase has been proposed as an important mediator of inflammation [29]. In this study, WLP elicits an anti-inflammatory response through the L-arginine-NO pathway. In addition, COX inhibitors have been widely used clinically. In recent years, several COX-2 selective inhibitors have been developed to reduce severe gastric side effects. In RAW264.7 cells, we demonstrate that WLP inhibits COX-2 protein expression. This suggests that WLP could have an anti-inflammatory effect. 

The activation of iNOS is correlated with the inflammation because inflammatory molecules like TNF-*α* and IL-1*β* expressions are tremendously upregulated compared to the unstimulated tissue. TNF-*α* is a mediator of Carr-induced inflammatory incapacitation and is able to induce the further release of kinins and leukotrienes, which are suggested to play an important role in the maintenance of long-lasting nociceptive response [30]. This study found that WLP decreases the TNF-*α* level in serum following Carr injection (Figure 3(d)). Some researches demonstrate that inflammatory effect induced by Carr is associated with free radicals. Free radicals, prostaglandin, and NO will be released when administering Carr for 1–6 h [31]. In addition, there is significantly increase in CAT, SOD, and GPx activities in paw edema with WLP treatment. Further, there is a significant decrease in MDA level with WLP treatment (Figure 3(b)). This data implies that the anti-inflammatory effect of WLP could be due at least in part to elevated intracellular antioxidant enzyme activities and decreased oxidative stress in tissue. In other words, the decreased lipid oxidation is probably due to the increased CAT, SOD, and GPx activities.

## 5. Conclusions

Our experimental study revealed that a WLP can inhibit production of oxidation, elevated antioxidant enzyme activities, and decreased inflammatory response. The anti-inflammatory effects of WLP may be related to NO and tumor necrosis factor *α* (TNF-*α*) suppression and associated with the increase in the activities of antioxidant enzymes, including catalase, superoxide dismutase, and glutathione peroxidase. Overall, the results suggest that longan pericarp may have the potential to be developed as a natural antioxidant or inflammatory inhibitor.

## Figures and Tables

**Table 1:** The effects of WLP on radical scavenging, reducing activity and liposome protection.

Tests	WLP (mg/mL)		
	0.05	0.1	0.2
Total polyphenols (*μ*g/mL)	11.7 ± 0.4^a^	18.5 ± 0.3^b^	29.2 ± 0.5^c^
ABTS inhibition (%)	46.6 ± 2.6^a^	91.3 ± 2.5^b^	94.8 ± 0.7^b^
DPPH inhibition (%)	36.0 ± 2.8^a^	70.0 ± 0.5^b^	96.7 ± 3.7^c^
Reducing activity (OD_700_)	0.230 ± 0.033^a^	0.423 ± 0.020^b^	0.499 ± 0.004^c^
Liposome protection (%)	12.6 ± 0.1^a^	20.6 ± 5.6^b^	37.0 ± 3.8^c^

Results are displayed with mean ± SEM (*n* = 3). Values with different superscripts in each row are significantly different (*P* < 0.05).

**Table 2:** The effects of WLP and three markers on DPPH assay and inhibition of NO production in LPS-induced RAW264.7 macrophages.

Samples	DPPH-scavenging activity *( *EC_50_, *μ*g/mL)	NO inhibition (IC_50_, *μ*g/mL)
WLP	69.4 ± 0.5	179.8 ± 1.4
Gallic acid	3.5 ± 0.1	18.8 ± 0.3
Epicatechin	26.2 ± 0.1	>100
Ellagic acid	2.8 ± 0.1	5.2 ± 0.2
Indomethacin	Not determined	52.5 ± 2.7

All values expressed as mean ± SEM (*n* = 3). Means which did not share a common letter were significantly different (*P* < 0.05) when analyzed by ANOVA and Ducan's multiple-range tests.

**Table 3:** Effects of WLP and indomethacin (Indo) on changes in CAT, SOD, and GPx activities in Carr-induced paw edema (5th hr) in mice.

Groups	CAT (U/mg protein)	SOD (U/mg protein)	GPx (U/mg protein)
Control	4.49 ± 0.42	3.54 ± 0.14	24.19 ± 0.14
Carr	2.73 ± 0.27^###^	1.52 ± 0.12^###^	17.40 ± 0.27^###^
Carr + Indo	3.86 ± 0.43*	2.45 ± 0.06*	20.12 ± 0.18*
Carr + WLP (100 mg/kg)	3.30 ± 0.18	1.94 ± 0.17	19.28 ± 0.38
Carr + WLP (200 mg/kg)	3.64 ± 0.21*	2.03 ± 0.06	20.79 ± 0.07*
Carr + WLP (400 mg/kg)	3.93 ± 0.07**	2.32 ± 0.01*	21.98 ± 0.08**

Each value represents as mean ± SEM. ^###^ *P* < 0.001 as compared with the control. *P < 0.05 and **P < 0.01 as compared with the Carr group.
